# Supplementary material for: Prevalence of Hepatitis E Virus (HEV) in Feral and Farmed Wild Boars in Xinjiang, Northwest China
Source: Viruses. 2022 Dec 27;15(1):78. doi: 10.3390/v15010078 (PMC9867238; doi:10.3390/v15010078)
Supplement: Supplementary file 1 [file viruses-15-00078-s001.zip › viruses-2015785-supplementary.pdf]

Table S1. Hepatitis E virus (HEV) RNA positivity in feral and farmed wild boars by age

		HEV RNA (fecal)				HEV RNA (liver)			
Age (months)	No. tested	No. positive	%	95% CI	p-value	No. positive	%	95% CI	p-value
Feral wild boar*					1.000				0.010
0–6	8	0	0.0	(0.0–36.9)		0#	0.0	(0.0–84.2)	
7–12	43	1	2.3	(0.0–12.3)		5#	29.4	(10.3–56.0)	
13–24	10	0	0.0	(0.0–30.8)		1	10.0	(0.3–44.5)	
25–36	25	0	0.0	(0.0–13.7)		0	0.0	(0.0–13.7)	
>36	35	0	0.0	(0.0–10.0)		1	2.9	(0.0–14.9)	
Farmed wild boar					0.701				
0–3	66	2	3.0	(0.3–10.5)		-			
4–6	194	5	2.6	(0.8–5.9)		-			
7–9	100	4	4.0	(1.1–9.9)		-			
10–12	102	1	1.0	(0.0–5.3)		-			
>12	16	0	0	(0.0–20.6)		-			

* Age was roughly estimated based on body weight, tusks, and neck mane.

# We failed to collect liver samples from 6 and 26 feral wild boars of age 0-6 months and 7-12 months respectively.
